# Targeting Endothelial Cell-Specific Molecule 1 Protein in Cancer: A Promising Therapeutic Approach

**DOI:** 10.3389/fonc.2021.687120

**Published:** 2021-05-24

**Authors:** He Zhang, Yi-Wen Shen, Li-Jun Zhang, Jin-Jiao Chen, Hui-Ting Bian, Wen-Jie Gu, Hong Zhang, Hong-Zhuan Chen, Wei-Dong Zhang, Xin Luan

**Affiliations:** ^1^ Institute of Interdisciplinary Integrative Medicine Research, Shanghai University of Traditional Chinese Medicine, Shanghai, China; ^2^ School of Pharmacy, Fudan University, Shanghai, China; ^3^ School of Medicine, Shanghai Jiao Tong University, Shanghai, China; ^4^ School of Pharmacy, Second Military Medical University, Shanghai, China

**Keywords:** endothelial cell-specific molecule 1, endocan, cancer, cellular function, tumor microenvironment, targeted therapy

## Abstract

Despite the dramatic advances in cancer research in the past few years, effective therapeutic strategies are urgently needed. Endothelial cell-specific molecule 1 (ESM-1), a soluble dermatan sulfate proteoglycan, also known as endocan, serves as a diagnostic and prognostic indicator due to its aberrant expression under pathological conditions, including cancer, sepsis, kidney diseases, and cardiovascular disease. Significantly, ESM-1 can promote cancer progression and metastasis through the regulation of tumor cell proliferation, migration, invasion, and drug resistant. In addition, ESM-1 is involved in the tumor microenvironment, containing inflammation, angiogenesis, and lymph angiogenesis. This article reviews the molecular and biological characteristics of ESM-1 in cancer, the underlying mechanisms, the currently clinical and pre-clinical applications, and potential therapeutic strategies. Herein, we propose that ESM-1 is a new therapeutic target for cancer therapy.

## Introduction

With the development of molecular oncology and the advance of cancer diagnosis and treatment, there is a substantial reduction in both incidence and mortality (1). However, cancer is still one of the most challenging diseases in clinical practice, and effective therapeutic strategies are urgently needed. A comprehensive understanding of the molecular mechanism associated with cancer is essential for further development. In particular, some proteins play a pivotal role in cancer development and their expression levels are related to cancer diagnosis and prognosis (2). Endothelial cell-specific molecule 1 (ESM-1), also known as endocan, is a soluble dermatan sulfate (DS) proteoglycan. It is secreted by various cell lines, especially by human vascular endothelial cells, and can be detected in the human bloodstream (3).

Accidentally discovered by French scientist Lassalle in 1996, ESM-1 was considered to be restricted in endothelial cells, therefore, it was named endothelial cell-specific molecule 1 (4). As ESM-1 is a kind of DS proteoglycan secreted specifically by endothelial cells, belonging to the PGs family, David Bechard renamed ESM-1 to endocan in 2001 (3).

DS is a glycosaminoglycan (GAG) which is produced through the epimerization of the glucuronic acid on chondroitin sulfate into iduronic acid by dermatan sulfate epimerase (DS-epi) 1 and 2. DS plays vital roles during the process of tumorigenesis, which is attributed to the increased flexibility of the polysaccharide chain in the presence of iduronic acid residues, facilitating specific interactions with proteins like growth factors, cytokines, and angiogenic factors (5, 6). As a kind of soluble DS proteoglycan, ESM-1 plays an important role in cancer initiation and progression. ESM-1 takes part in molecular interactions in a wide range of biological progresses, including cell proliferation, adhesion, migration, and invasion (7, 8). Moreover, ESM-1 has been implicated in vascular tissue development under normal or pathological conditions and it is often used as an indicator of angiogenesis in clinical practice. Studies have shown that ESM-1 is aberrantly expressed in a myriad of diseases, such as cancer, vascular disorder, and inflammation (9). Recently, ESM-1 has garnered immense attention owing to its distinctive role in tumorigenesis and tumor progression. In this review, we discussed the multifaceted role of ESM-1 in some important cellular processes relevant to cancer and the underlying mechanisms. The current therapeutic approaches targeting at ESM-1 are also comprehensively summarized.

## The Structure, Expression, and Regulation of ESM-1

**Figure 1:**
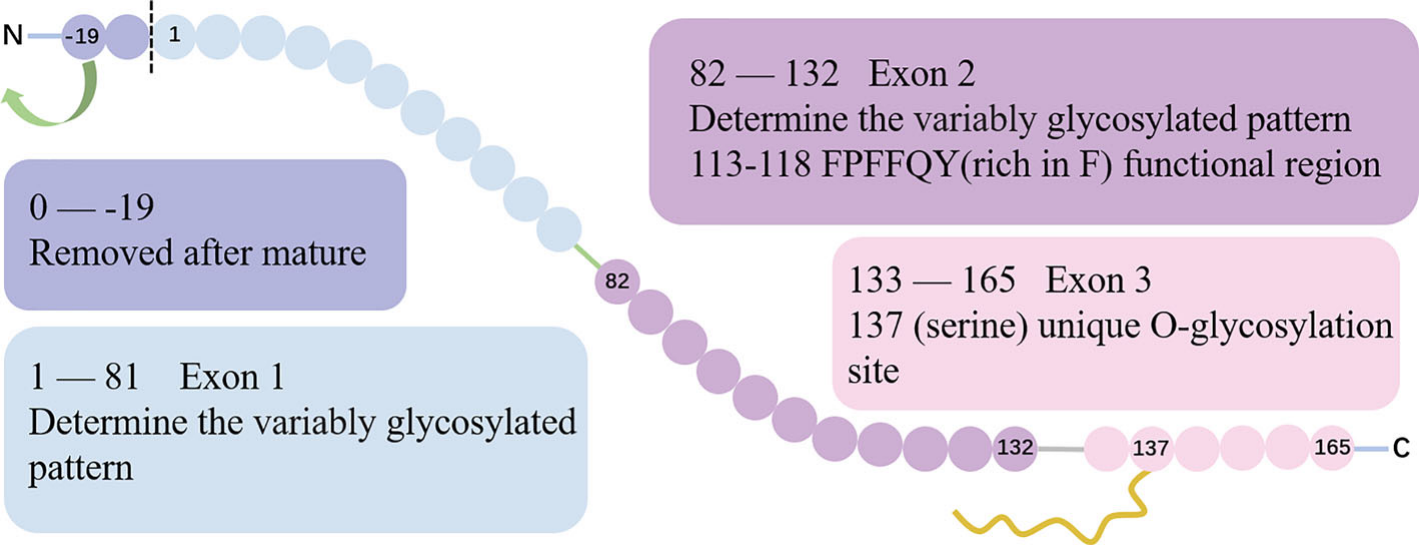
The gene structure and protein sequence of ESM-1. The gene structure of ESM-1. Exon 1 and part of exon 2 encode for an N-terminal cysteine-rich region. The exon 2 encodes a special region rich in F (113FPFFQY118), which is the functional region of ESM-1. The boundary between exon 1 and exon 2 encoding is the site of 82. Exon 3 encodes for a short 33 amino-acid long C-terminal region that includes the unique O-glycosylation site at serine 137.

Located on chromosome 5 at the position q11.2, ESM-1 gene contains 3 exons and 2 introns with a total length of 2006 base pairs. Exon 1 and a part of exon 2 encode for a N-terminal cysteine-rich region of 110 amino acids residues. Besides, exon 2 encodes for the functional region rich in phenylalanine (F, ^113^FPFFQY^118^) (10). Finally, exon 3 encodes for a short 33 amino-acid long C-terminal region that features the O-glycosylation site at serine 137 (3, 4, 11). The gene structure and protein sequence of ESM-1 is show in (**Figure 1**).

The initial coding protein of ESM-1 contains 184 amino acids. With the removal of the hydrophobic signal peptide sequence which is composed of 19 amino acid residues at the N-terminal, a mature protein containing 165 amino acids is formed. Taken together, ESM-1 is a 50 kDa cysteine-rich proteoglycan composed of 165 amino acids mature protein cores (20 kDa) and approximately 30 kDa corresponding to a unique dermatan sulfate chain linked to serine residues after being post-translationally modified (12, 13).

Originally, as ESM-1 is found at endothelial cells of lung tissue, scientists thought that ESM-1 is highly restricted to the lung vascular endothelial cells (4). Further studies revealed that ESM-1 also expressed in kidney, liver, lung, thyroid gland, lymph nodes, skin, and gastrointestinal tract (3, 13, 14). However, ESM-1 is not detected in vascular-rich organs such as heart, pancreas, and placenta (15).

The expression level of ESM-1 can be up-regulated by a multitude of proinflammatory cytokines and growth factors, such as tumor necrosis factor-α (TNF-α), interleukin-1β (IL-1β), interleukin-8 (IL-8), E-selectin, transforming growth factors-β1 (TGF-β1), lipopolysaccharide (LPS), nuclear factor-κB (NF-κB), phorbol myristate acetate (PMA), and retinoic acid (4, 13, 16–19). Vascular endothelial growth factor (VEGF) is a significant modulator which is capable of up-regulating the expression of ESM-1. Under the treatment of VEGF *in vitro*, expression of mRNA levels of ESM-1 is elevated in a time and dose-dependent manner (15). Interestingly, VEGF impacts on ESM-1 transcription through PKC-NF-κB signaling pathway, instead of Src or MEK/MAPK pathway (15, 20). In addition, ESM-1 is significant elevated in intermittent hypoxia (IH) compared to normoxia (21). IH plays a vital role in ESM-1 expression *via* HIF-1α/VEGF pathway. Under the condition of IH, HIF-1α is dramatically upregulated, and subsequently stimulates the expression of VEGF which is a positive regulator of ESM-1 (21).

In contrast, ESM-1 can be down-regulated by interleukin-4 (IL-4), interferon-γ (IFN-γ), phosphatidylinositol 3-kinase (PI3K), and homeodomain transcription factor (HHEX) (20, 22, 23). HHEX, a proline-rich homologous domain protein belonging to the homeobox family, can directly inhibit ESM-1 gene by binding to HRE which is the original HHEX responder in *ESM-1* gene (24).

## The Role of ESM-1 in Cancer

**Figure 2:**
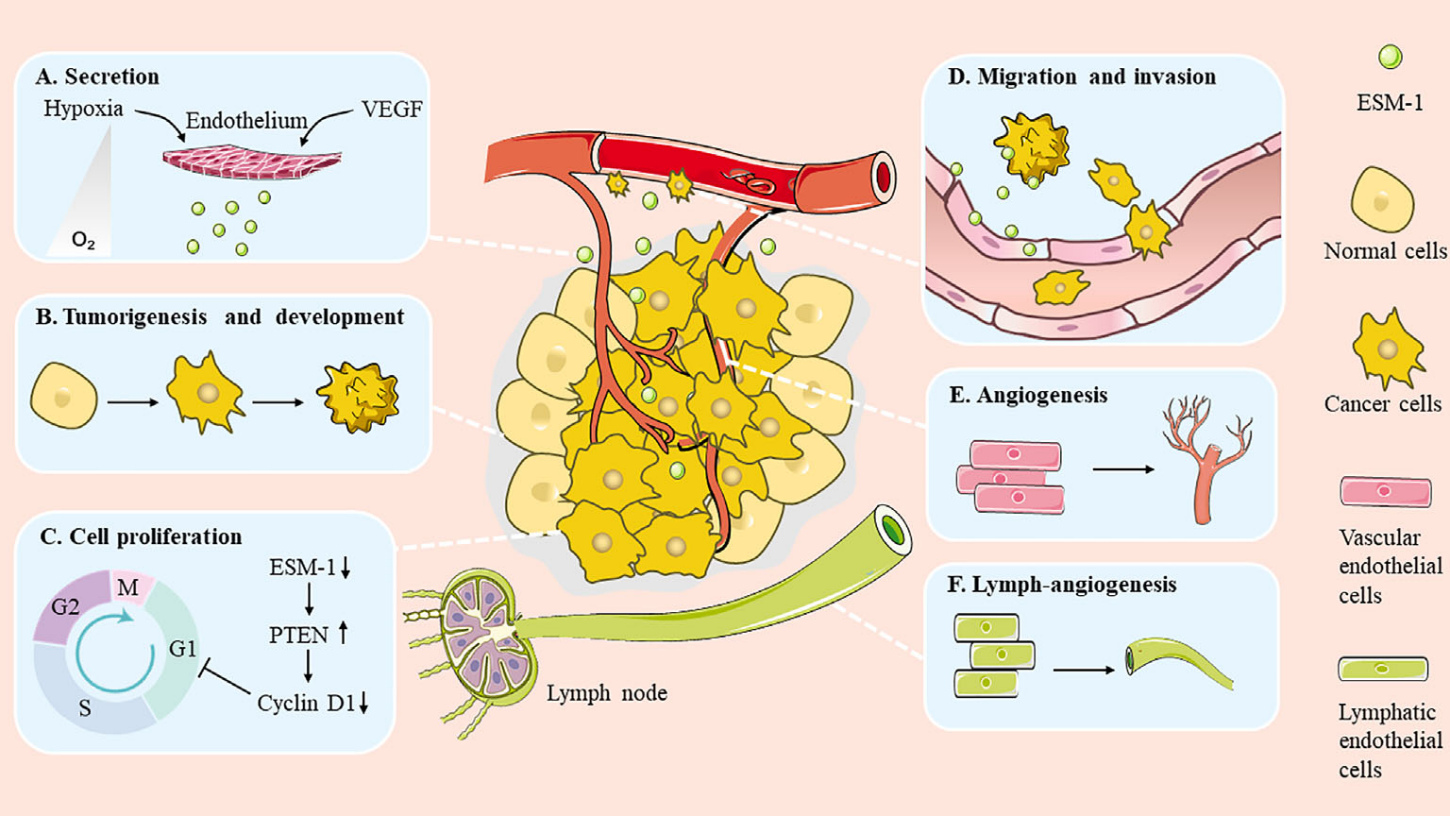
Role of ESM-1 during cancer progression. ESM-1 participates in multiple biological progresses in the cancer development. **(A)** Secretion. **(B)** Tumorigenesis and development. **(C)** Cell proliferation. **(D)** Migration and invasion. **(E)** Angiogenesis. **(F)** Lymph-angiogenesis.

Emerging evidence indicates that ESM-1 expression is elevated in a broad spectrum of cancers. It is also worth noting that the expression level of ESM-1 is related to tumor aggressiveness and tumor vascularization (8). In recent years, substantial investigations have focused on the relationship between ESM-1 and cancer. It has been found that ESM-1 is involved in many aspects of tumorigenesis, such as promoting cell cancerization, modulating cell proliferation and survival, as well as regulating migration and invasion (**Figure 2**). In addition, ESM-1 is closely related to drug resistance.

### Promoting Cell Cancerization

Studies have shown that overexpression of ESM-1 in non-tumorigenic epithelial cells can induce tumor formation, while overexpression of ESM-1 in tumorigenic cells can significantly increase the growth rate of tumors (20, 25). Carcinogenesis of ESM-1 requires the existence of glycosylation chain and the integral protein structure, because specific protein mutations can lead to the loss of tumor promoting effect. The activity of ESM-1 in promoting tumor cell growth also depends on the protein core of ESM-1, especially the phenylalanine rich region centered on amino acid residues 115 and 116 (25). Yassine et al. in 2008 conducted a study on the role of ESM-1 in tumor progression in mice, showing that overexpression or systemic administration of endogenous non-glycosylated ESM-1 could delay the growth of HT-29 tumor cells. They believed that since human fully glycosylated ESM-1 can promote tumor growth, there is an interesting hypothesis that the balance between glycosylated ESM-1 and non-glycosylated ESM-1 can induce or delay tumor growth (10). Furthermore, ESM-1 can facilitate the transformation of dormant tumors into fast-growing angiogenic types and induce tumor formation (23, 24). However, ESM-1 protein does not participate in fibroblast growth factor 2 (FGF-2) induced endothelial cell proliferation, because it is not inhibited by anti-ESM-1 protein antibodies (25). Therefore, the cellular target of ESM-1 seems to be malignant epithelial cells rather than endothelium itself. It is speculated that ESM-1 protein plays a role in the paracrine positive feedback loop and facilitates tumor growth. Meanwhile, ESM-1 protein in turn plays a role in amplifying the effect of tumor growth factors and inhibiting the migration of effective immune cells into the tumor (4, 26).

### Modulating Cell Proliferation and Survival

Previous studies have shown that ESM-1 can affect cell proliferation and cell survival. NF-κB is always secreted aberrantly in cancers, influencing the processes of oncogeneses such as cell proliferation and apoptosis, metastasis, and angiogenesis. On the basis of in-depth studies regarding the underlying mechanisms, researchers attributed the suppression of cell proliferation to the cell cycle arrest, which is a result of the regulation of NF-κB and PTEN by ESM-1 (26). Yun and his colleagues have found that knockdown of ESM-1 decreased the expression of NF-κB in colorectal cancer and hepatocellular cancer. In addition, PTEN, which plays a vital role in G1 cell cycle arrest, is activated after silencing ESM-1 and results in cell cycle protein cyclin D1 reduction and localization in the nucleus (27, 28).

Additionally, researchers found that the cell viability of the colorectal cancer cell lines COLO205 and SK-HEP1 was reduced up to 15% and 11% respectively after ESM-1 silencing (27, 28). Subsequent studies unraveled that ESM-1 increased tumor cell survival rate *via* the Akt-dependent NF-κB/IκB pathway. When the ESM-1 gene silenced, phospho-Akt and NF-κB subfamily members like NF-κB p65 were down-regulated and the phospho-IκBα was up-regulated, while the expression level of phospho-JNK or ERK1/2 was not altered. Interestingly, phospho-p38 MAPK was also decreased but phospho-Akt expression was more strongly suppressed than phospho-p38 MAPK expression in ESM-1 gene silencing cells compared to control. Taken together, JNK, ERK, and p38 pathways are not directly related to the suppression of survival (27, 28).

### Regulation of Migration and Invasion

Cell migration and invasion are essential elements of cancer metastasis, which is responsible for nearly 90% of cancer-associated mortality (29). An interesting observation is that ESM-1 mRNA level is much higher in metastatic tumor samples than in non-metastatic tumor samples, indicating that ESM-1 expression is associated with metastasis (28). Moreover, ESM-1 is closely related to the vascular invasion. In gastric cancer, researchers found that tumors with ESM-1 overexpression show more easily invasion into vascular, causing more frequently distant metastasis (30).

During cancer invasion and metastasis, the surrounding basement membrane can be destroyed by expression of the transcript levels of matrix metalloproteinases (MMPs), making tumor cells drop from the primary focus and spread to new tissues. A recent study has shown that migration- and metastasis-associated proteins, such as MMP-9 were decreased by ESM-1 regulation and recombinant human (Rh) TIMP-1, a significant MMP-9 inhibitor, was increased by ESM-1 regulation. However, the ESM-1 mediated cell migration and invasion were enhanced. This may be attribute to the imbalance of MMP-9/TIMP-1, which is the mechanism of ESM-1 promoting metastasis of prostate cancer cells (31). ESM-1 expression significantly affects the migration of cells in colorectal, gastric, nasopharyngeal, head and neck cancer, and hepatocellular carcinomas (27, 28, 31, 32).

When silencing ESM-1 *via* siRNA, levels of NF-κB, phospho-Akt and glycogen synthase kinase 3 (GSK3α/β) are markedly decreased, whereas levels of E-cadherin are elevated. Akt and GSK3 have been proved to be involved in metastasis. GSK3 relates to the actin cytoskeleton, microtubules, and adhesion turnover in diverse stages of cell migration. These results indicate that ESM-1 expression may be related to cell migration and invasion (27, 31).

### Drug Resistance

Traditional chemotherapy and emerging molecularly targeted therapies have significantly improved the efficacy of cancer therapeutics. However, drug resistance remains a major obstacle for cancer treatment, resulting to tumor cells insensitive to chemotherapeutic drugs and/or other anti-cancer drugs. Usually, the development of drug resistance involves a series of gene and protein interactions (33, 34). Previous studies have revealed that *ESM-1* is one of drug resistance-related genes (35). In patients with prolactinoma, ESM-1-microvessel density in bromocriptine (which belongs to dopamine agonist to treat prolactinoma) resistant patients was dramatically higher than that of sensitive groups. However, blocking ESM-1 with siRNAs markedly increased the sensitivity of rat prolactinoma cell lines GH3 and MMQ to dopamine agonist treatment (35). Similar observation has been made in HUVEC cells. Silencing of ESM-1 significantly increased the sensitivity of HUVECs to bevacizumab to treatment (35).

## The Role of ESM-1 in Tumor Microenvironment

Tumors are dynamic entities which grow in a highly complex milieu called tumor microenvironment (TME). TME encompasses not only tumor cells, but also multiple other components like immune cells, endothelial cells, adipocytes, fibroblasts, extracellular matrix (ECM), as well as blood and lymph vessels (36). The intricate microenvironment affects tumor growth, invasion, and metastasis. Mounting evidence indicates that targeting TME may provide a reliable alternative to conventional tumor-targeted therapy (37).

### ESM-1 and Inflammation

Inflammation is strongly associated with cancer. There is a growing body of evidence indicating that inflammation contributes to the development and progression of malignancies (38, 39), and it is regarded as one of hallmarks of cancer (40). It has been demonstrated that ESM-1 can influence leukocyte adhesion and extravasation, which are essential for inflammatory processes. Researches show that ESM-1 can reduce leukocyte adhesion by directly binding to leukocyte function-associated antigen-1 (LFA-1) *in vitro* (41). LFA-1, an integrin molecule, interacts with its most important ligand intercellular adhesion molecule-1 (ICAM-1), promoting leukocytes adhesion to endothelial cells (42, 43). ESM-1 has been found to directly bind to LFA-1 on the cell surface of leukocytes, and antagonize interaction with endothelial-cell expressed ICAM-1, reducing leukocyte adhesion *in vitro* (41). However, leukocyte-to-endothelial cell adhesion was not reduced by ESM-1 in a biomimetic microfluidic assay by Zheng et al. (44). This data suggests that LFA-1 is not the only adhesion molecule on leukocytes that binds to ICAM-1 (44). To further investigate the impact of ESM-1 on leukocyte *in vivo*, researchers performed peritonitis assays on ESM-1 knockout mice and wild type mice. ESM-1 knockout mice showed a significant decrease in leukocyte extravasation compared with wild mice, indicating ESM-1 plays an important role in leukocyte extravasation (45). Moreover, researchers used the intravital microscopy to direct visualization and analysis of leukocyte behavior during the rolling, adhesion, and transmigration steps. They found that the impaired leukocyte extravasation in ESM-1 knockout mice was attributed to reduced transmigration. Immunostainings for leukocyte-endothelial cells adherens and tight junctions show that there are no significant structural differences between ESM-1 knockout mice and wild type mice (45). Therefore, targeting ESM-1 could be a promising strategy to treat inflammation and cancer.

### Regulation of Angiogenesis

Tumor vascularization is a hallmark of cancer and has been shown as an important step in cancer progression and metastasis (46). The VEGF pathway is one of the key mediators of angiogenesis in cancer. As mentioned above, VEGF-A is a specific inducer of ESM-1 transcription which is supported by the fact that ESM-1 expression is up-regulated in VEGF-A treated endothelial cells (20). Notably, emerging studies have identified that ESM-1 is also implicated in angiogenesis (**Figure 2**). ESM-1 is overexpressed during *in vitro* angiogenesis. However, ESM-1 seems not to modify angiogenesis directly (17), as HUVEC tube formation induced by VEGF and FGF-2 did not change after ESM-1 knockdown (47). Mechanistic investigations on the role of ESM-1 in angiogenesis revealed that there is a positive feedback loop between VEGF-A and ESM-1. ESM-1 expression was stimulated by VEGF-A through the phosphorylation and activation of VEGFR-2 (48). In turn, ESM-1 binds to fibronectin directly and thereby displaces fibronectin-bound VEGF-A_165_, resulting in increased bioavailability of VEGF-A_165_ and subsequently enhanced VEGF-A mediated signaling (45). Consistent with this, researchers found that more VEGF-A_165_ is sequestered to fibronectin and decreases VEGF bioavailability and signaling after ESM-1 knockdown (45). Moreover, it has been reported that ESM-1 is enriched in specialized tip cells and is associated with filopodia (49–51). The tip cells are one of the motile endothelial cells, which mediate the sprouting of developing vessels during the process of angiogenesis (52). Filopodia are typically present in tip cells and are needed for their guidance and motility during angiogenesis. In ESM-1 knockout mice retina models, retinal vascular outgrowth and filopodia emission are impaired. In further studies, F. Rocha et al. found that filopodia number is significantly decreased after ESM-1 knockdown and the impaired vascular progression might be due to the reduction of motility of endothelial sprouts (45).

In addition, ESM-1 can bind to HGF/SF, FGF-2 and other pro-angiogenic molecules, exerting the effect of angiogenesis promotion. The DS chain of ESM-1 can bind to HGF/SF and FGF-2 and promote the proliferation of vascular endothelial cells, resulting in neovascularization. ESM-1 and HGF/SF, FGF-2 can promote the expression of each other in the form of positive feedback regulation, which makes vascular endothelial cell proliferation more obvious and easier to form tubular structure (4).

### Regulation of Lymph Angiogenesis

Tumor lymphatic vessels execute complex functions during cancer progression. Metastatic tumor cells readily invade permeable peritumoral lymphatic vessels and metastasis (53). Moreover, tumors can induce lymph angiogenesis within their draining lymph nodes even before metastasis and induce lymph angiogenesis in lymph nodes, promoting the further metastasis to distant lymph nodes and organs (12).

Previous studies have shown that the growth and activation of lymphatic vessels can be mediated by VEGF-C and/or VEGF-A. Of note, ESM-1 can be potently induced by both VEGF-C and VEGF-A during lymph angiogenesis (**Figure 2**). A follow-up study revealed that incubation of lymphatic endothelial cells (LECs) with ESM-1 enhanced the stimulating effects of both VEGF-A and VEGF-C on LEC proliferation and migration, whereas incubation with ESM-1 alone did not have the same effect. However, gene silencing of ESM-1 significantly inhibited the induction of LEC proliferation and migration by VEGF-A and/or VEGF-C. Taken together, ESM-1 induced by VEGF-A and/or VEGF-C represents an autocrine, positive feedback loop which further promote the stimulatory functions of both growth factors on lymphatic endothelial cells. Moreover, ESM-1 induction by VEGF-A was mainly dependent on the activation of VEGFR-2, as inhibition of VEGFR-2 completely inhibits the VEGF-A mediated induction of ESM-1 in LECs. And VEGF-C mediated induction depended on the activity of both VEGFR-2 and VEGFR-3 (12). In brief, ESM-1 can enhance the function of other lymphangiogenic factors (such as VEGF-A, VEGF-C) to indirectly promote lymph angiogenesis and tumor metastasis. It can be a novel mediator of lymph angiogenesis and regarded as a potential target for the inhibition of VEGF-A– or VEGF-C–induced pathologic lymphatic vessel activation.

## Clinical Relevance of ESM-1 in Cancers

ESM-1 protein is aberrantly expressed in many cancers, including hematologic and solid tumors (**Table 1**). The over- or under-expression of ESM-1 compared to normal tissues is related to worse clinicopathologic features and clinical outcomes, indicating the ESM-1 as a biomarker for diagnosis and prognosis.

**Table 1:** ESM-1 expression in human tumors.

Cancers	Research sample	Expression level	Clinical characterization	Reference
Bladder Cancer	Tissue	High	Shorter recurrence-free survival time in noninvasive bladder cancers	(48, 54)
Breast cancer (TNBC)	Plasma	High	Poor outcome	(55)
Clear cell renal cell carcinoma	Serum	High	Poor Survival	(56)
Colorectal cancer	Saliva	High	Poor prognosis, high histological differentiation, high depth of tumor invasion high TNM stage and high lymph node metastasis	(57, 58)
	Tissue	Lower	Poor differentiate	(59)
Ovarian cancer	Tissue	High	Poor survival	(60)
	Serum	High	More malignancy	(61)
Gastric cancer	Tissue	High	Poor survival Distant and lymph nodes metastasis Vascular invasion	(30, 62)
	Serum	High	Poor progonsis	(63)
Hepatocellular Carcinoma	Tissue	High	Poor Survival	(64)
	Serum	High	High tumor stage	(28)
Meningiomas and Gliomas	Tissue	High	High degree of malignancy	(65)
Multiple myeloma	Plasma	High	Higher disease stage	(66)
Non-small cell lung cancer	Tissue	High	Poor prognosis and distant metastasis	(17)
	Pleural effusion	High	Poor prognosis, poor survival and distant metastasis	(67)
Oral cancer	Plasma	High	Associated with tumor (T) status, higher at T1-T3 status	(68)
Pancreatic neuroendocrine tumors	Tissue	High	Poor clinical outcomes and greater malignancy	(69)
Pituitary adenoma	Tissue	High	Associated with Knosp tumor invasion grades, higher recurence, more aggressive and invasion	(70–72)
Prostate cancer	Serum	High	Easily recurrence	(73)
	Tissue	High	Higher Gleason grades and scores	(74)

TNBC, Triple-negative breast cancer; TNM, Tumor node metastasis.

As ESM-1 is a secreted protein, it is a potential marker for cancer. In gastric cancer, higher serum ESM-1 level is related to poor prognosis (63). In non-small cell lung cancer, ESM-1 level is higher in malignant pleural effusion (MPE) compared with benign pleural effusion (BPE), and is associated with poor prognosis and distant metastasis (67). ESM-1 is also an indicator for the aggressive behavior of cancer. Although barely expressed in endothelial cells of normal pituitary, ESM-1 is highly expressed in endothelial and/or endocrine cells in patients with pituitary adenomas (70). In addition, the higher ESM-1 level indicates bigger tumor size, raised mitotic count and elevated p53 expression (70). In gastric cancer, ESM-1 is strikingly overexpressed in tumor tissue, resulting in tumor proliferation, distant lymph nodes metastasis, as well as vascular invasion (30, 62). In addition, ESM-1 is related to Borrmann type IV which is an independent prognosis factor for survival in gastric cancer (30, 75).

Interestingly, ESM-1 is only enhanced in triple negative breast cancer (TNBC), the most aggressive and easily metastatic breast cancer subtype, and its expression is positively correlated with the malignancy of cancer. MDA-MB-231BR is a brain metastatic variant of human TNBC cell line MDA-MB-231 and is more malignant than MDA-MB-231 cells. ESM-1 is strikingly overexpressed in MDA-MB-231BR, which might be due to DNA demethylation in an upstream region of *ESM-1* gene (55).

Although there are a series of clinicopathologic parameters aiming different cancers, novel and effective biomarkers are still urgently needed to improve the clinical prediction of cancer progression and recurrence. In addition, thoroughly understanding the relationship between ESM-1 expression and the subsistent clinicopathological characters of cancers is helpful for combining diagnosis. In pituitary adenomas, ESM-1 is positively correlated to Knosp tumor invasion grades, which is useful to estimate the degree of invasion by ESM-1 expression (71). In multiple myeloma, ESM-1 is elevated in plasma and is associated with disease stage, hypercalcemia, renal failure, anemia, and bone lesions. The additional secretion of ESM-1 may attribute to the secretion of VEGF by malignant plasma cells and the interaction with bone marrow microenvironment (66). In prostate cancer, ESM-1 expression is significantly elevated in patients with high Gleason grades and Gleason scores, the main method used to stage prostate cancer, suggesting ESM-1 is a useful biomarker for cancer diagnosis (74).

In addition, ESM-1 is a potential parameter to monitor the tumor response to anti-angiogenic therapeutics. An interesting observation in clear cell renal cell carcinoma is that sunitinib can prevent VEGF-induced ESM-1 secretion by endothelial cells, but not regulate the TNF-α-induced ESM-1 secretion (56), suggesting that ESM-1 could be a potential biomarker to monitor the patient response to anti-VEGF therapies (56).

Angiogenesis is crucial for cancer progression and is an indicator for poor prognosis. It is worthy to note that ESM-1 is also a biomarker of neovascularization, the hallmark of tumor development, invasion, and metastasis (40). Angiogenesis is always evaluated by tumor microvascular density (MVD) that is counting CD-31 or CD-34 antibodies positive vessels (60). Nevertheless, the major defect is that these antibodies will mark endothelial cells of both normal and tumor tissues (76). Therefore, it is necessary to find a highly specific marker to label the endothelium of tumor tissue but not with the endothelium of most normal tissue. As mentioned above, ESM-1 is over-expressed in tumor tissues but under-expressed in normal tissues. Mounting evidence indicates that the MVD denoted by ESM-1 (ESM-1-MVD) has prognostic value in malignancy that higher ESM-1-MVD is correlated with a shorter survival time. In addition, ESM-1-MVD was strongly associated with tumor histology, grading, tumor size, and clinical staging, and has been proven to be one of the most accurate prognostic indicators of postoperative recurrence in ovarian cancer (60), gastric cancer (76, 77), and hepatocellular carcinoma (64). Taken together, ESM-1-MVD is a useful marker for prognosis, which can identify those who might benefit from a different follow-up approach and subsequent adjuvant therapy.

## Therapeutic Strategies Targeting ESM-1 in Cancer

Given the intimate relationship between ESM-1 and tumor biology, ESM-1 promises to be an CD11a/CD18 active target in cancer therapy. Tremendous efforts have been made or underway to explore the potential value of modulating ESM-1 for cancer treatment in experimental and clinical settings. However, there are no small molecular drugs targeting ESM-1 in clinic currently. This may be attributed to the indeterminacy of ESM-1 protein crystal structure. Hence, there are still many difficulties in developing drugs targeting ESM-1.

### Exon 2 Deletion of ESM-1 Could be a Future Cancer Therapy Target

The biologic functions of proteoglycans often depend on the interactions of their glycosaminoglycan chains with protein ligands, such as cytokines and growth factors (78, 79). As ESM-1 is a kind of proteoglycans which is involved in tumor development, the glycosylation chain is essential for its biological activity. Researchers demonstrate that the tumorigenic property of ESM-1 is highly dependent on its glycan chain. In addition, the protein core and a Phenylalanine-rich (F-rich) region situated between residues F113 and F116 are also critical (80).

Depontieu et al. identified exon 2 sequence is closely connected with protein multimerization, the glycation status, and the cancerogenic activity of ESM-1. Deletion of exon 2 related amino acid sequence (called ESM-1Δ2) impaired the synthesis of glycan chain, and ESM-1Δ2 loss the function of cancerization. Moreover, various cell lines of human and murine origin were implanted in the skin of severe combined immunodeficient mice, and ESM-1Δ2 did not promote tumor formation. These suggesting that exon 2-derived sequence may represent a future therapeutic target against cancer (80).

### Silencing ESM-1 by RNAi and Antibody

#### siRNA and shRNA

The high expression and sustained activation of ESM-1 is prevailing in tumor cells and ESM-1 is recognized as a highly dependent gene for survival. RNA interference technology, a highly functional tool for researchers to prevent pathogenic gene expression, was mainly used to study the function of a gene or disease (81).

A broad array of genetic knockdown experiments validated the potential of ESM-1 as a powerful target for clinical evaluation in the treatment of cancer. Blocking ESM-1 with small interfering RNAs (siRNAs) successfully inhibited tumor cell proliferation, migration, invasion, and angiogenesis in diverse cancers (**Table 2**). Moreover, ESM-1 knockdown with short hairpin RNAs (shRNAs) dramatically inhibit the NGFR induced tumor growth, invasion, and metastasis in oral squamous cell carcinoma (84).

**Table 2:** List of therapeutic strategies targeting ESM-1.

Intervention	Cancer type	Results	Reference
siRNA	Colorectal Cancer	Inhibit migration,invasion and decrease cell survival	(26, 54)
	Hepatocellular carcinoma	Decrease cell survival, migration, and invasion and modulated cell cycle progression	(28)
	Head and neck cancer	Inhibit proliferation, migration	(81)
	Invasive Bladder Cancer	Inhibit VEGF-A–induced tube formation, migration, and VEGFR-2 phosphorylation	(48)
	Gastric cancer	Inhibit proliferation	(62)
	Proatate cancer	Inhibit migration	(73, 82)
	Triple-negative breast cancer	Inhibit proliferation, colony, migration, invasion	(83)
shRNA	Oral squamous cell carcinoma	Inhabit NGFR-induced the tumor growth, invasion, and metastasis	(84)
Antibody (PABsc-20343)	Gastric cancer	Inhibit tumor cells and vascular endothelial cells proliferation	(29)
miR-9-3p	Bladder cancer	Inhibit viability, migration, and invasion, and promote apoptosis	(85)

VEGF-A, Vascular endothelial growth factor A; VEGFR-2, Vascular endothelial growth factor receptor 2; NGFR, Nerve growth factor receptor.

Despite the initially encouraging ESM-1 knockdown outcomes *in vivo*, most reports utilized the gene silencing techniques in cultured cells, and less is known about how malignant cells response to blockade agents in established tumors. The research and development of RNAi targeting ESM-1 have great potential in the future.

#### miRNA

MicroRNAs (miRNAs) are endogenous, small non-coding RNAs which contain about 20 to 25 nucleotides, and play regulatory roles in target gene expression by complexing with RNA‐induced silencing complexes (RISCs) and interacting with the 3’ untranslated regions (3’UTRs) of target mRNAs (85–87). Since ESM-1 are upregulated in many cancers, interference with ESM-1 by miRNA may be a potential therapeutic strategy.

As reported, miR-9-3p, derived from bone marrow-derived mesenchymal stem cells (BMSCs) secreted exosomes, can target gene *ESM-1* to inhibit viability, migration, and invasion while promoting apoptosis in bladder cancer cells. Additionally, ESM-1 is down-regulation after exosomal miR-9-3p treatment, and the cancer progression and metastasis are both prevented in xenograft model of bladder cancer in mice (88).

Despite miRNA therapeutic strategy is a promising treatment approach to cancer, there are several questions remain to be addressed. MiRNA is a multiple targeted regulator which often regulates multiple mRNAs, as mRNA recognition only requires binding to the 5’- end of the seed region of miRNA rather than the entire nucleotide sequence (89, 90). Hence, it is essential to develop a method to deliver miRNA to tumor cells at the target site, which can improve cell specificity. Besides, it is necessary to develop effective delivery systems to protect miRNA escaping degraded by serum nucleases, removed by immune system, and to increase the target cell uptake.

#### Antibody

With the approval of bevacizumab (Avastin) and cetuximab (Erbitux), antibody-based therapeutics appear to be vital components of therapies for multiple cancers (91). Although ESM-1 is a kind of secreted protein which is suitable for monoclonal antibody blockade, the research on anti-ESM-1 antibody is limited. In gastric cancer, ESM-1 overexpressed endothelial cells HMEC-1 and adenocarcinoma cells MKN28 are treated with a goat polyclonal antibody (PABsc-20343) to antagonize ESM-1. As a result, there is a marked reduction of cell numbers, suggesting that down-regulation of ESM-1 can inhibit tumor cells and vascular endothelial cells proliferation (30).

## Conclusions

ESM-1 is an endothelial dysfunction marker and participates in diverse endothelium-dependent pathological disorders, including cancer, sepsis, kidney diseases and cardiovascular disease (25, 92, 93). Herein, we have attempted to shed light on the unique role of ESM-1 in cancers. It is worth noting that ESM-1 is not only intrinsically promoting tumor growth, but also regulating tumor microenvironment. In addition, ESM-1 is a biomarker for diagnosis and prognosis of cancers.

Given the significant role of ESM-1 in cancers, it may be a potential target in cancer treatment, and the inhibitors targeting ESM-1 are urgently needed. Hindered by lack of precise ESM-1 crystal structure, the design-based small-molecule inhibitor discovery and identification, has lagged. The utilization of advanced molecular biology techniques, such as RNAi, to target ESM-1 have shown encouraging results. However, these methods are only used *in vitro* or in animal models, due to the failure to reach the target organs or tissues specifically. Nanocarriers provide us with a novel delivery system to protect RNAs and antibodies from degradation in blood vessels, deliver them to target organs, and promote intracellular accumulation. Taken together, further studies are required to completely clarify the regulations of ESM-1 and the underlying mechanisms under different physiological conditions.

## Author Contributions

W-DZ, XL contributed to conceptualization, review and editing, funding acquisition, and supervision. HeZ and Y-WS carried out the original draft, made figures and wrote the paper. L-JZ, J-JC, H-TB, W-JG, HoZ, and H-ZC revised the paper. All authors contributed to the article and approved the submitted version.

## Funding

This work was supported by the National Natural Science Foundation of China (No.81903654), Program for Professor of Special Appointment (Young Eastern Scholar) at Shanghai Institutions of Higher Learning (QD2018035), Shanghai Chenguang Program (No.18CG46), Shanghai Sailing Program (19YF1449400), Shanghai Engineering Research Centre for the Preparation of Bioactive Natural Products (16DZ2280200), the National Key Research and Development Program of China (2017YFC1700200), and National Major Project of China (2019ZX09201004-003-010).

## Conflict of Interest

The authors declare that the research was conducted in the absence of any commercial or financial relationships that could be construed as a potential conflict of interest.

## References

[1] Rong BX Cai XG Liu H Yang SY. Stathmin-Dependent Molecular Targeting Therapy for Malignant Tumor: The Latest 5 Years’discoveries and Developments. J Transl Med (2016) 14:297. 10.1186/s12967-016-1000-z 27670291 PMC5037901

[2] Rong B Yang S. Molecular Mechanism and Targeted Therapy of Hsp90 Involved in Lung Cancer: New Discoveries and Developments. Int J Oncol (2018) 52(2):321–36. 10.3892/ijo.2017.4214 29207057

[3] Béchard D Gentina T Delehedde M Scherpereel A Lyon M Aumercier M. Endocan is a Novel Chondroitin Sulfate/Dermatan Sulfate Proteoglycan That Promotes Hepatocyte Growth Factor/Scatter Factor Mitogenic Activity. J Biol Chem (2001) 276(51):48341–9. 10.1074/jbc.M108395200 11590178

[4] Lassalle P Molet S Janin A Van der Heyden J Tavernier J Fiers W. ESM-1 is a Novel Human Endothelial Cell-Specific Molecule Expressed in Lung and Regulated by Cytokines. J Biol Chem (1996) 271(34):20458–64. 10.1074/jbc.271.34.20458 8702785

[5] Yamada S Sugahara K. Potential Therapeutic Application of Chondroitin Sulfate/Dermatan Sulfate. Curr Drug Discov Technol (2008) 5(4):289–301. 10.2174/157016308786733564 19075609

[6] Listik E Xavier EG Silva PM Toma L. Dermatan Sulfate Epimerase 1 Expression and Mislocalization may Interfere With Dermatan Sulfate Synthesis and Breast Cancer Cell Growth. Carbohydr Res (2020) 488:107906. 10.1016/j.carres.2020.107906 31972438

[7] Kali A Shetty KS. Endocan: A Novel Circulating Proteoglycan. Indian J Pharmacol (2014) 46(6):579–83. 10.4103/0253-7613.144891 PMC4264070 25538326

[8] Abu EA Nawaz MI De Hertogh G Al-Kharashi AS Van den Eynde K Mohammad G. The Angiogenic Biomarker Endocan is Upregulated in Proliferative Diabetic Retinopathy and Correlates With Vascular Endothelial Growth Factor. Curr Eye Res (2015) 40(3):321–31. 10.3109/02713683.2014.921312 24871583

[9] Sarrazin S Adam E Lyon M Depontieu F Motte V Landolfi C. Endocan or Endothelial Cell Specific Molecule-1 (ESM-1): A Potential Novel Endothelial Cell Marker and a New Target for Cancer Therapy. Biochim Biophys Acta (2006) 1765(1):25–37. 10.1016/j.bbcan.2005.08.004 16168566

[10] Scherpereel A Gentina T Grigoriu B Senechal S Janin A Tsicopoulos A. Overexpression of Endocan Induces Tumor Formation. Cancer Res (2003) 63(18):6084–9. 14522939

[11] Sarrazin S Lyon M Deakin JA Guerrini M Lassalle P Delehedde M. Characterization and Binding Activity of the Chondroitin/Dermatan Sulfate Chain From Endocan, a Soluble Endothelial Proteoglycan. Glycobiology (2010) 20(11):1380–8. 10.1093/glycob/cwq100 20581009

[12] Shin JW Huggenberger R Detmar M. Transcriptional Profiling of VEGF-A and VEGF-C Target Genes in Lymphatic Endothelium Reveals Endothelial-Specific Molecule-1 as a Novel Mediator of Lymphangiogenesis. Blood (2008) 112(6):2318–26. 10.1182/blood-2008-05-156331 PMC2532805 18614759

[13] Bechard D Meignin V Scherpereel A Oudin S Kervoaze G Bertheau P. Characterization of the Secreted Form of Endothelial-Cell-Specific Molecule 1 by Specific Monoclonal Antibodies. J Vasc Res (2000) 37(5):417–25. 10.1159/000025758 11025405

[14] Zhang SM Zuo L Zhou Q Gui SY Shi R Wu Q. Expression and Distribution of Endocan in Human Tissues. Biotech Histochem (2012) 87(3):172–8. 10.3109/10520295.2011.577754 21526908

[15] Abid MR Yi X Yano K Shih S Aird WC. Vascular Endocan is Preferentially Expressed in Tumor Endothelium. Microvasc Res (2006) 72(3):136–45. 10.1016/j.mvr.2006.05.010 16956626

[16] Scherpereel A Depontieu F Grigoriu B Cavestri B Tsicopoulos A Gentina T. Endocan, a New Endothelial Marker in Human Sepsis. Crit Care Med (2006) 34(2):532–7. 10.1097/01.ccm.0000198525.82124.74 16424738

[17] Grigoriu BD Depontieu F Scherpereel A Gourcerol D Devos P Ouatas T. Endocan Expression and Relationship With Survival in Human Non-Small Cell Lung Cancer. Clin Cancer Res (2006) 12(15):4575–82. 10.1158/1078-0432.CCR-06-0185 16899604

[18] Kaplanski G Fabrigoule M Boulay V Dinarello CA Bongrand P Kaplanski S. Thrombin Induces Endothelial Type II Activation *In Vitro*: IL-1 and TNF-Alpha-Independent IL-8 Secretion and E-selectin Expression. J Immunol (1997) 158(11):5435–41. 9164965

[19] Kirwan RP Leonard MO Murphy M Clark AF O’Brien CJ. Transforming Growth Factor-β-Regulated Gene Transcription and Protein Expression in Human GFAP-Negative Lamina Cribrosa Cells. Glia (2005) 52(4):309–24. 10.1002/glia.20247 16078232

[20] Rennel E Mellberg S Dimberg A Petersson L Botling J Ameur A. Endocan is a VEGF-A and PI3K Regulated Gene With Increased Expression in Human Renal Cancer. Exp Cell Res (2007) 313(7):1285–94. 10.1016/j.yexcr.2007.01.021 17362927

[21] Sun H Zhang H Li K Wu H Zhan X Fang F. ESM-1 Promotes Adhesion Between Monocytes and Endothelial Cells Under Intermittent Hypoxia. J Cell Physiol (2019) 234(2):1512–21. 10.1002/jcp.27016 30144067

[22] Maurage CA Adam E Mineo JF Sarrazin S Debunne M Siminski RM. Endocan Expression and Localization in Human Glioblastomas. J Neuropathol Exp Neurol (2009) 68(6):633–41. 10.1097/NEN.0b013e3181a52a7f 19458546

[23] Cong R Jiang X Wilson CM Hunter MP Vasavada H Bogue CW. Hhex is a Direct Repressor of Endothelial Cell-Specific Molecule 1 (ESM-1). Biochem Bioph Res Co (2006) 346(2):535–45. 10.1016/j.bbrc.2006.05.153 16764824

[24] Pellizzari L D’Elia A Rustighi A Manfioletti G Tell G Damante G. Expression and Function of the Homeodomain-Containing Protein Hex in Thyroid Cells. Nucleic Acids Res (2000) 28(13):2503–11. 10.1093/nar/28.13.2503 PMC102703 10871399

[25] De Freitas CN Gaudet A Portier L Tsicopoulos A Mathieu D Lassalle P. Endocan, Sepsis, Pneumonia, and Acute Respiratory Distress Syndrome. Crit Care (2018) 22(1):280. 10.1186/s13054-018-2222-7 30367649 PMC6204032

[26] Rasmi RR Sakthivel KM Guruvayoorappan C. NF-kappaB Inhibitors in Treatment and Prevention of Lung Cancer. BioMed Pharmacother (2020) 130:110569. 10.1016/j.biopha.2020.110569 32750649

[27] Kang YH Ji NY Han SR Lee CI Kim JW Yeom YI. ESM-1 Regulates Cell Growth and Metastatic Process Through Activation of NF-kappaB in Colorectal Cancer. Cell Signal (2012) 24(10):1940–9. 10.1016/j.cellsig.2012.06.004 22735811

[28] Kang YH Ji NY Lee CI Lee HG Kim JW Yeom YI. ESM-1 Silencing Decreased Cell Survival, Migration, and Invasion and Modulated Cell Cycle Progression in Hepatocellular Carcinoma. Amino Acids (2011) 40(3):1003–13. 10.1007/s00726-010-0729-6 20821239

[29] Seyfried TN Huysentruyt LC. On the Origin of Cancer Metastasis. Crit Rev Oncog (2013) 18(1-2):43–73. 10.1615/critrevoncog.v18.i1-2.40 23237552 PMC3597235

[30] Liu N Zhang L Du H Hu Y Zhang G Wang X. Overexpression of Endothelial Cell Specific Molecule-1 (ESM-1) in Gastric Cancer. Ann Surg Oncol (2010) 17(10):2628–39. 10.1245/s10434-010-1037-9 20383661

[31] Chen CM Lin CL Chiou HL Hsieh SC Lin CL Cheng CW. Loss of Endothelial Cell-Specific Molecule 1 Promotes the Tumorigenicity and Metastasis of Prostate Cancer Cells Through Regulation of the TIMP-1/MMP-9 Expression. Oncotarget (2017) 8(8):13886–97. 10.18632/oncotarget.14684 PMC5355147 28108731

[32] Sumei Z Shaolong C Xiang W Yinliang Q Qing Z Yuan W. Endocan Reduces the Malign Grade of Gastric Cancer Cells by Regulating Associated Protein Expression. Tumor Biol (2016) 37(11):14915–21. 10.1007/s13277-016-5398-y 27644250

[33] Tolios A De Las RJ Hovig E Trouillas P Scorilas A Mohr T. Computational Approaches in Cancer Multidrug Resistance Research: Identification of Potential Biomarkers, Drug Targets and Drug-Target Interactions. Drug Resist Updat (2020) 48:100662. 10.1016/j.drup.2019.100662 31927437

[34] Assaraf YG Brozovic A Goncalves AC Jurkovicova D Line A Machuqueiro M. The Multi-Factorial Nature of Clinical Multidrug Resistance in Cancer. Drug Resist Updat (2019) 46:100645. 10.1016/j.drup.2019.100645 31585396

[35] Cai L Leng ZG Guo YH Lin SJ Wu ZR Su ZP. Dopamine Agonist Resistance-Related Endocan Promotes Angiogenesis and Cells Viability of Prolactinomas. Endocrine (2016) 52(3):641–51. 10.1007/s12020-015-0824-2 26662185

[36] Di Martino JS Mondal C Bravo-Cordero JJ. Textures of the Tumour Microenvironment. Essays Biochem (2019) 63(5):619–29. 10.1042/EBC20190019 PMC6839695 31654075

[37] Hanna E Quick J Libutti SK. The Tumour Microenvironment: A Novel Target for Cancer Therapy. Oral Dis (2009) 15(1):8–17. 10.1111/j.1601-0825.2008.01471.x 18992016

[38] Dupre A Malik HZ. Inflammation and Cancer: What a Surgical Oncologist Should Know. Eur J Surg Oncol (2018) 44(5):566–70. 10.1016/j.ejso.2018.02.209 29530345

[39] Grivennikov SI Greten FR Karin M. Immunity, Inflammation, and Cancer. Cell (2010) 140(6):883–99. 10.1016/j.cell.2010.01.025 PMC2866629 20303878

[40] Hanahan D Weinberg RA. Hallmarks of Cancer: The Next Generation. Cell (2011) 144(5):646–74. 10.1016/j.cell.2011.02.013 21376230

[41] Béchard D Scherpereel A Hammad H Gentina T Tsicopoulos A Aumercier M. Human Endothelial-Cell Specific Molecule-1 Binds Directly to the Integrin CD11a/CD18 (LFA-1) and Blocks Binding to Intercellular Adhesion Molecule-1. J Immunol (2001) 167(6):3099–106. 10.4049/jimmunol.167.6.3099 11544294

[42] Zimmerman T Blanco F. Inhibitors Targeting the LFA-1/ICAM-1 Cell-Adhesion Interaction: Design and Mechanism of Action. Curr Pharm Des (2008) 14(22):2128–39. 10.2174/138161208785740225 18781967

[43] Helena Yusuf-Makagiansar MEAT. Inhibition of LFA-1/ICAM-1 and VLA-4/VCAM-1 as a Therapeutic Approach to Inflammation and Autoimmune Diseases. Med Res (2002) 22(2):146–7. 10.1002/med.10001 11857637

[44] Zheng X Soroush F Long J Hall ET Adishesha PK Bhattacharya S. Murine Glomerular Transcriptome Links Endothelial Cell-Specific Molecule-1 Deficiency With Susceptibility to Diabetic Nephropathy. PloS One (2017) 12(9):e185250. 10.1371/journal.pone.0185250 PMC5608371 28934365

[45] Rocha SF Schiller M Jing D Li H Butz S Vestweber D. Esm1 Modulates Endothelial Tip Cell Behavior and Vascular Permeability by Enhancing VEGF Bioavailability. Circ Res (2014) 115(6):581–90. 10.1161/CIRCRESAHA.115.304718 25057127

[46] Donovan P Patel J Dight J Wong HY Sim SL Murigneux V. Endovascular Progenitors Infiltrate Melanomas and Differentiate Towards a Variety of Vascular Beds Promoting Tumor Metastasis. Nat Commun (2019) 10(1):18. 10.1038/s41467-018-07961-w 30604758 PMC6318267

[47] Aitkenhead M Wang S Nakatsu MN Mestas J Heard C Hughes CCW. Identification of Endothelial Cell Genes Expressed in an *In Vitro* Model of Angiogenesis: Induction of ESM-1, βig-h3, and NrCAM. Microvasc Res (2002) 63(2):159–71. 10.1006/mvre.2001.2380 11866539

[48] Roudnicky F Poyet C Wild P Krampitz S Negrini F Huggenberger R. Endocan is Upregulated on Tumor Vessels in Invasive Bladder Cancer Where it Mediates VEGF-A-induced Angiogenesis. Cancer Res (2013) 73(3):1097–106. 10.1158/0008-5472.CAN-12-1855 23243026

[49] Recchia FM Xu L Penn JS Boone B Dexheimer PJ. Identification of Genes and Pathways Involved in Retinal Neovascularization by Microarray Analysis of Two Animal Models of Retinal Angiogenesis. Invest Ophthalmol Vis Sci (2010) 51(2):1098–105. 10.1167/iovs.09-4006 PMC2868453 19834031

[50] Strasser GA Kaminker JS Tessier-Lavigne M. Microarray Analysis of Retinal Endothelial Tip Cells Identifies CXCR4 as a Mediator of Tip Cell Morphology and Branching. Blood (2010) 115(24):5102–10. 10.1182/blood-2009-07-230284 20154215

[51] Del TR Prahst C Mathivet T Siegfried G Kaminker JS Larrivee B. Identification and Functional Analysis of Endothelial Tip Cell-Enriched Genes. Blood (2010) 116(19):4025–33. 10.1182/blood-2010-02-270819 PMC4314527 20705756

[52] Haupt F Krishnasamy K Napp LC Augustynik M Limbourg A Gamrekelashvili J. Retinal Myeloid Cells Regulate Tip Cell Selection and Vascular Branching Morphogenesis *Via* Notch Ligand Delta-like 1. Sci Rep (2019) 9(1):9798. 10.1038/s41598-019-46308-3 31278348 PMC6611798

[53] Petrova TV Koh GY. Biological Functions of Lymphatic Vessels. Science (2020) 369(6500):eaax4063. 10.1126/science.aax4063 32646971

[54] Laloglu E Aksoy H Aksoy Y Ozkaya F Akcay F. The Determination of Serum and Urinary Endocan Concentrations in Patients With Bladder Cancer. Ann Clin Biochem (2016) 53(6):647–53. 10.1177/0004563216629169 26748103

[55] Sagara A Igarashi K Otsuka M Kodama A Yamashita M Sugiura R. Endocan as a Prognostic Biomarker of Triple-Negative Breast Cancer. Breast Cancer Res Treat (2017) 161(2):269–78. 10.1007/s10549-016-4057-8 PMC5225208 27888420

[56] Leroy X Aubert S Zini L Franquet H Kervoaze G Villers A. Vascular Endocan (ESM-1) Is Markedly Overexpressed in Clear Cell Renal Cell Carcinoma. Histopathology (2010) 56(2):180–7. 10.1111/j.1365-2559.2009.03458.x 20102396

[57] Ji NY Kim Y Jang YJ Kang YH Lee CI Kim JW. Identification of Endothelial Cell-Specific Molecule-1 as a Potential Serum Marker for Colorectal Cancer. Cancer Sci (2010) 101(10):2248–53. 10.1111/j.1349-7006.2010.01665.x PMC11158300 20735430

[58] Jiang H Fu XG Chen YT. Serum Level of Endothelial Cell-Specific Molecule-1 and Prognosis of Colorectal Cancer. Genet Mol Res (2015) 14(2):5519–26. 10.4238/2015.May.25.3 26125749

[59] Zuo L Zhang S Hu R Zhu H Zhou Q Gui S. Correlation Between Expression and Differentiation of Endocan in Colorectal Cancer. World J Gastroentero (2008) 14(28):4562. 10.3748/wjg.14.4562 PMC2731287 18680240

[60] El Behery MM Seksaka MA Ibrahiem MA Saleh HS El Alfy Y. Clinicopathological Correlation of Endocan Expression and Survival in Epithelial Ovarian Cancer. Arch Gynecol Obstet (2013) 288(6):1371–6. 10.1007/s00404-013-2863-3 23708323

[61] Laloglu E Kumtepe Y Aksoy H Topdagi YE. Serum Endocan Levels in Endometrial and Ovarian Cancers. J Clin Lab Anal (2017) 31(5):e22079. 10.1002/jcla.22079 PMC6816907 27734523

[62] Zhao W Sun M Li S Wang Y Liu J. Biological and Clinical Implications of Endocan in Gastric Cancer. Tumor Biol (2014) 35(10):10043–9. 10.1007/s13277-014-2287-0 25012244

[63] Lv Z Fan Y Chen H Zhao D. Endothelial Cell-Specific Molecule-1: A Potential Serum Marker for Gastric Cancer. Tumor Biol (2014) 35(10):10497–502. 10.1007/s13277-014-2319-9 25056533

[64] Huang G Tao Y Ding X. Endocan Expression Correlated With Poor Survival in Human Hepatocellular Carcinoma. Digest Dis Sci (2009) 54(2):389–94. 10.1007/s10620-008-0346-3 18592377

[65] Atukeren P Kunbaz A Turk O Kemerdere R Ulu MO Turkmen IN. Expressions of Endocan in Patients With Meningiomas and Gliomas. Dis Markers (2016) 2016:7157039. 10.1155/2016/7157039 27528791 PMC4978841

[66] Steiner N Hajek R Sevcikova S Borjan B Untergasser G Gobel G. The Plasma Levels of the Angiogenic Cytokine Endocan are Elevated in Patients With Multiple Myeloma. Anticancer Res (2018) 38(9):5087–92. 10.21873/anticanres.12828 30194153

[67] Lu GJ Shao CJ Zhang Y Wei YY Xie WP Kong H. Diagnostic and Prognostic Values of Endothelial-Cell-Specific Molecule-1 With Malignant Pleural Effusions in Patients With Non-Small Cell Lung Cancer. Oncotarget (2017) 8(30):49217–23. 10.18632/oncotarget.17455 PMC5564762 28514746

[68] Yang WE Hsieh MJ Lin CW Kuo CY Yang SF Chuang CY. Plasma Levels of Endothelial Cell-Specific Molecule-1 as a Potential Biomarker of Oral Cancer Progression. Int J Med Sci (2017) 14(11):1094–100. 10.7150/ijms.20414 PMC5666540 29104463

[69] Lin L Yeh Y Chu C Won JGS Shyr Y Chao Y. Endocan Expression is Correlated With Poor Progression-Free Survival in Patients With Pancreatic Neuroendocrine Tumors. Medicine (2017) 96(41):e8262. 10.1097/MD.0000000000008262 29019897 PMC5662320

[70] Cornelius A Cortet-Rudelli C Assaker R Kerdraon O Gevaert MH Prevot V. Endothelial Expression of Endocan Is Strongly Associated With Tumor Progression in Pituitary Adenoma. Brain Pathol (2012) 22(6):757–64. 10.1111/j.1750-3639.2012.00578.x PMC8057624 22353248

[71] Matano F Yoshida D Ishii Y Tahara S Teramoto A Morita A. Endocan, a New Invasion and Angiogenesis Marker of Pituitary Adenomas. J Neuro Oncol (2014) 117(3):485–91. 10.1007/s11060-014-1377-6 24504498

[72] Miao Y Zong M Jiang T Yuan X Guan S Wang Y. A Comparative Analysis of ESM-1 and Vascular Endothelial Cell Marker (CD34/CD105) Expression on Pituitary Adenoma Invasion. Pituitary (2016) 19(2):194–201. 10.1007/s11102-015-0698-6 26809958 PMC4799238

[73] Arslan B Onuk Ö Hazar 0 Aydın M Çilesiz NC Eroglu A. Prognostic Value of Endocan in Prostate Cancer: Clinicopathologic Association Between Serum Endocan Levels and Biochemical Recurrence After Radical Prostatectomy. Tumori J (2017) 103(2):204–8. 10.5301/tj.5000535 27470607

[74] Lai C Chen C Hsu W Hsieh Y Liu C. Overexpression of Endothelial Cell-Specific Molecule 1 Correlates With Gleason Score and Expression of Androgen Receptor in Prostate Carcinoma. Int J Med Sci (2017) 14(12):1263–7. 10.7150/ijms.21023 PMC5666560 29104483

[75] An JY Kang TH Choi MG Noh JH Sohn TS Kim S. Borrmann Type IV: An Independent Prognostic Factor for Survival in Gastric Cancer. J Gastrointest Surg (2008) 12(8):1364–9. 10.1007/s11605-008-0516-9 18516653

[76] Folkman J Klagsbrun M. Angiogenic Factors. Science (1987) 235(4787):442–7. 10.1126/science.2432664 2432664

[77] Chang Y Niu W Lian P Wang X Meng Z Liu Y. Endocan-Expressing Microvessel Density as a Prognostic Factor for Survival in Human Gastric Cancer. World J Gastroentero (2016) 22(23):5422. 10.3748/wjg.v22.i23.5422 PMC4910663 27340359

[78] Hardingham TE Fosang AJ. Proteoglycans: Many Forms and Many Functions. FASEB J (1992) 6(3):861–70. 10.1096/fasebj.6.3.1740236 1740236

[79] Bishop JR Schuksz M Esko JD. Heparan Sulphate Proteoglycans Fine-Tune Mammalian Physiology. Nature (2007) 446(7139):1030–7. 10.1038/nature05817 17460664

[80] Depontieu F Grigoriu BD Scherpereel A Adam E Delehedde M Gosset P. Loss of Endocan Tumorigenic Properties After Alternative Splicing of Exon 2. BMC Cancer (2008) 8:14. 10.1186/1471-2407-8-14 18205914 PMC2254430

[81] Bender O Gunduz M Cigdem S Hatipoglu OF Acar M Kaya M. Functional Analysis of ESM1 by siRNA Knockdown in Primary and Metastatic Head and Neck Cancer Cells. J Oral Pathol Med (2018) 47(1):40–7. 10.1111/jop.12648 29024069

[82] Rebollo J Geliebter J Reyes N. Esm-1 siRNA Knockdown Decreased Migration and Expression of CXCL3 in Prostate Cancer Cells. Int J BioMed Sci (2017) 13(1):35–42. 28533735 PMC5422643

[83] Jin H Rugira T Ko YS Park SW Yun SP Kim HJ. ESM-1 Overexpression is Involved in Increased Tumorigenesis of Radiotherapy-Resistant Breast Cancer Cells. Cancers (2020) 12(6):1363. 10.3390/cancers12061363 PMC7352712 32466580

[84] Chen C Shin JH Eggold JT Chung MK Zhang LH Lee J. ESM1 Mediates NGFR-induced Invasion and Metastasis in Murine Oral Squamous Cell Carcinoma. Oncotarget (2016) 7(43):70738–49. 10.18632/oncotarget.12210 PMC5342586 27683113

[85] Di Leva G Garofalo M Croce CM. MicroRNAs in Cancer. Annu Rev Pathol (2014) 9:287–314. 10.1146/annurev-pathol-012513-104715 24079833 PMC4009396

[86] Esquela-Kerscher A Slack FJ. Oncomirs - microRNAs With a Role in Cancer. Nat Rev Cancer (2006) 6(4):259–69. 10.1038/nrc1840 16557279

[87] Lujambio A Lowe SW. The Microcosmos of Cancer. Nature (2012) 482(7385):347–55. 10.1038/nature10888 PMC3509753 22337054

[88] Cai H Yang X Gao Y Xu Z Yu B Xu T. Exosomal microRNA-9-3p Secreted From BMSCs Downregulates ESM1 to Suppress the Development of Bladder Cancer. Mol Ther Nucleic Acids (2019) 18:787–800. 10.1016/j.omtn.2019.09.023 31734559 PMC6861677

[89] Ahmadzada T Reid G McKenzie DR. Fundamentals of siRNA and miRNA Therapeutics and a Review of Targeted Nanoparticle Delivery Systems in Breast Cancer. Biophys Rev (2018) 10(1):69–86. 10.1007/s12551-017-0392-1 29327101 PMC5803180

[90] Thomson DW Bracken CP Goodall GJ. Experimental Strategies for microRNA Target Identification. Nucleic Acids Res (2011) 39(16):6845–53. 10.1093/nar/gkr330 PMC3167600 21652644

[91] Adams GP Weiner LM. Monoclonal Antibody Therapy of Cancer. Nat Biotechnol (2005) 23(9):1147–57. 10.1038/nbt1137 16151408

[92] Nalewajska M Gurazda K Marchelek-Myśliwiec M Pawlik A Dziedziejko V. The Role of Endocan in Selected Kidney Diseases. Int J Mol Sci (2020) 21(17):6119. 10.3390/ijms21176119 PMC7504273 32854332

[93] Balta S Mikhailidis DP Demirkol S Ozturk C Celik T Iyisoy A. Endocan: A Novel Inflammatory Indicator in Cardiovascular Disease? Atherosclerosis (2015) 243(1):339–43. 10.1016/j.atherosclerosis.2015.09.030 26448266

